# Protocol for assessing the structural architecture, integrity, and cellular composition of murine bone e*x vivo*

**DOI:** 10.1016/j.xpro.2025.103843

**Published:** 2025-05-24

**Authors:** Jonathan W. Lewis, Kathryn Frost, Georgiana Neag, Holly Adams, Emily Powell, Orla Gallagher, Ilaria Bellantuono, Amy J. Naylor, Helen M. McGettrick

**Affiliations:** 1 Department of Inflammation and Ageing, School of Infection, Inflammation and Immunology, College of Medicine and Health, University of Birmingham, Birmingham, UK; 2 Academic Unit of Bone Metabolism and Mellanby Centre for Bone Research, University of Sheffield, Sheffield, UK

**Keywords:** Health Sciences, Microscopy, Model Organisms

## Abstract

Understanding changes in murine bone is essential to comprehend bone homeostasis and the role of therapeutics in bone diseases. Here, we present a protocol to assess the structural architecture, integrity, and cellular composition of murine bone *ex vivo*. We describe steps for imaging and analyzing bone by micro-computed tomography (CT) and functionally testing bone strength through three-point bending. We then detail procedures for histology and analysis of calcein labeling.

For complete details on the use and execution of this protocol, please refer to Lewis et al.^1^

## Highlights

• Protocol for in-depth analysis of murine bone from one animal
• Steps for analyzing bone architecture and strength *ex vivo*
• Procedures for analysis of osteoblasts and osteoclast cell numbers in bone

## Abstract (editor highlights)

Publisher’s note: Undertaking any experimental protocol requires adherence to local institutional guidelines for laboratory safety and ethics.

## Graphical abstract

**Figure:**
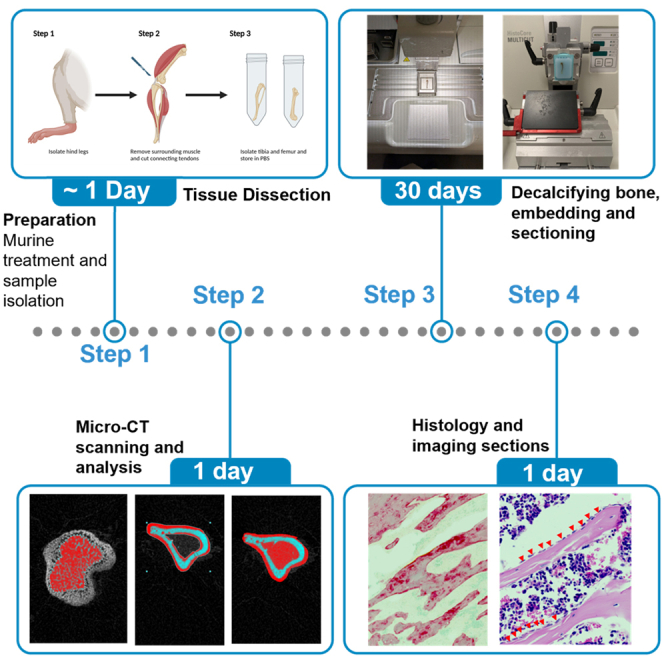

## Before you begin

This protocol describes the complete procedure for processing murine bones to analyze the structural architecture and integrity, as well as the cellular composition *ex vivo*. Before you begin to conduct animal experiments, please secure ethical approval to undertake animal research from your country/institutional. We recommend the use of the NC3Rs Experimental Design Assistant to design your animal experiments to ensure that they are appropriately powered to achieve the scientific objectives and to limit subjective bias within the scientific and analytical approaches.^2^ Additionally, refer to the key resources table, locating any equipment and preparing any reagents/solutions in advance as necessary.

### Institutional permissions

Animal studies were regulated by the Animals (Scientific Procedures) Act 1986 of the United Kingdom and performed under UK Home Office Personal Project License (PE5985209) at the Biomedical Services Unit, University of Birmingham, which holds a section 2C Establishment License. Approval was granted by the University of Birmingham’s Animal Welfare and Ethical Review Body, and all ethical guidelines were adhered to whilst carrying out this study.

### *In vivo* calcein labeling of murine bone

**Timing: 6+ days**

Prior to analysis of bone growth *ex vivo*, bones must be labeled on two different occasions with calcein. Calcein incorporates into the outer layer of bone following injection, thus bone growth over time can be analyzed by a further injection at a later time point.

1. Six days before the end point of live animal experiments, weigh out 12.0 mg calcein and combine with 3 mL deionized water (diH2O) (4 mg/mL) in a 5 mL sterilized Bijou container.
2. Vortex until fully dissolved.
3. Filter through a 0.22 μM sterile filter using a sterile syringe.

***Note:*** Filtered calcein solution can be frozen at −20°C until use.

4. Mice are treated with 20 mg/kg of calcein - e.g. for a 20 g mouse, 100 μL is required.
  a. Take up the required volume + 10% into a syringe.
  b. Invert the syringe and pull down the stopper to take a small amount of air into the syringe.
  c. Flick the syringe to dislodge any air bubbles to the tip and slowly release the air until liquid begins to emerge from the needle and the volume displays the amount required for injection.
5. Inject the calcein solution intra-peritoneally into the mouse.
6. Two days prior to collection of tissues repeat steps 1–5.

### Collection of murine tibias and femurs

**Timing: 1 h**

7. On day of collection prepare 2 labeled bijou tubes per mice containing ice cold sterile αMEM base media.
8. Euthanize the mouse using schedule 1 method as according to institutional permissions.
9. Using sterile surgical scissors, cut the skin above the peritoneum.
  a. Make a small incision above the peritoneum.
  b. With the scissors angled up, cut up towards the head, keeping the peritoneum intact.
  c. Cut around the mouse to separate the skin from the peritoneum.
10. Remove the skin from the legs,
  a. Make a cut around the paws and gently pull the skin to completely remove it.
11. Remove the hind limbs:
  a. Make an incision using a scalpel around the hip joint at the head of the femur. This should release the leg including the femur, tibia and foot.
    i. If it does not release easily gently twist the femoral head in the joint socket to release, being careful not to damage the tibia.
  b. Transfer samples into a labeled bijou tube and store on ice.
12. Clean all muscle from bone samples, using tweezers to gently remove the muscle surrounding the femur and tibia.
13. At the knee joint, make a small incision using a scalpel through the patella ligament. This should enable separation of the femur and tibia.
  ***Note:*** The ligament appears white and can be seen at the joint between the femur and tibia.
  a. When the ligament is fully severed it will be possible to completely bend the leg at the knee.
14. Fix samples for micro-CT analysis in 10% neutral buffered formalin overnight, to ensure bone from left and right limbs are identifiable place in separate tubes. Alternatively, bones used for 3-point bend testing can be directly frozen at −20°C for later analysis.
15. Following fixation, remove bones and wash in PBS three times.
  a. Add 5 mL of PBS to each sample.
  b. Remove PBS into waste container.
  c. Repeat steps 15a and b three times.
16. Transfer the samples to a Bijou and store in PBS + 0.02% sodium azide at 4°C before future analysis.

***Note:*** This method has been created to optimize the amount of data collected per mouse, allowing scanning, strength testing and imaging to be performed. In order to perform all techniques, the left or right tibia can be utilized for calcein labeling and micro-CT (micro-CT must be performed first), and the opposite side tibia can be utilized for the decalcification and staining protocols.

## Key resources table

**Table:** 

REAGENT or RESOURCE	SOURCE	IDENTIFIER
**Biological samples**		

Murine tibias	C57BL/6J male mice (8–12 weeks)	N/A
Murine femurs	C57BL/6J male mice (8–12 weeks)	N/A

**Chemicals, peptides, and recombinant proteins**		

Calcein	Sigma-Aldrich	Cat: C0875
10% buffered formaldehyde	Sigma-Aldrich	Cat: HT5011
Sodium azide	Sigma-Aldrich	Cat: S8032
α-MEM	Sigma-Aldrich	Cat: M8042
EDTA	Sigma-Aldrich	Cat: T4174
Ethanol	VWR	Cat: 20821
Xylene	Fisher Scientific	Cat: X/0250/17
Eosin	Pioneer Research Chemicals	Cat: PRC-661
Hematoxylin	Pioneer Research Chemicals	Cat: PRC-R51
Paraffin	Leica	Cat: 3801360
Fast Green	Sigma-Aldrich	Cat: F7252
Sirius Red	Sigma-Aldrich	Cat: 365548
Picric acid	Sigma-Aldrich	Cat: P6744
PBS	Oxoid	Cat: BR0014
LR White medium grade resin	TAAB Laboratories Equipment Ltd	Cat: L012
Sodium acetate anhydrous	VWR	Cat: 10236
L+ tartaric acid	Fisher Scientific	Cat: 137855000
Glacial acetic acid	Sigma-Aldrich	Cat: 1005706
Sodium hydroxide	Sigma-Aldrich	Cat: 221465
Naphthol AS-MX	Sigma-Aldrich	Cat: N4875
2-ethoxy ethanol	Alfa Aesar	Cat: 16100
Fast Red Violet LB salt	Sigma-Aldrich	Cat: F3381
ProLong Gold antifade mountant	Fisher Scientific	Cat: P36930

**Software and algorithms**		

NRecon 1.6.1.5	Bruker	N/A
DataViewer	Bruker	https://www.bruker.com/en/products-and-solutions/preclinical-imaging/micro-ct/3d-suite-software.html
CTAn v.1.12	Bruker	https://www.bruker.com/en/products-and-solutions/preclinical-imaging/micro-ct/3d-suite-software.html
Win-Test	Win-Test	http://www.win-test.com/
OsteoMeasure	OsteoMetrics	https://www.osteometrics.com/
ImageJ	National Institutes for Health	https://imagej.nih.gov/ij/
ImageJ Fiji	ImageJ	https://imagej.net/software/fiji/downloads
Zen Blue software	Zeiss	N/A

**Other**		

SkyScan 1172 micro-CT scanner	Bruker	N/A
ElectroForce BioDynamic 5500	Bose	N/A
Axioscan Z1	Zeiss	N/A
LSM 780 confocal microscope	Zeiss	N/A
LightCycler 480	Roche	Cat: 04729749001

## Step-by-step method details

### Micro-CT scanning of murine tibias

**Timing: 1 h per sample**

***Note:*** The following protocol is written for use with the SkyScan 1172 micro-CT machine, now discontinued, and murine bones. The methods discussed still apply to alternative micro-CT machines such as the updated version (Skyscan 1272 CMOS) and differing bone samples, however the X-ray voltage, current and exposure should be optimized for the specific machine and sample used, taking into account the age of both the machine and sample. Detailed and comprehensive guidelines for the assessment of bone microstructure (including mineral density, which was not performed here) in rodents using micro-computed tomography can be found in Bouxsein et al. 2010.^3^ In addition, guidelines for using other machines (e.g. Scanco micro-CT 40 machine) can be found elsewhere.^4^

**Figure 1:**
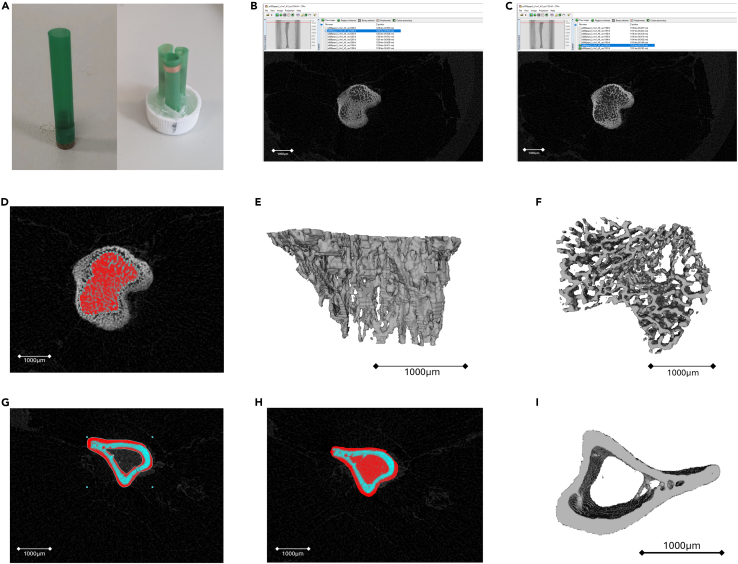
Generating regions of interest for micro-CT analysis (A) Exemplar methods to utilize straws for scanning of tibias by micro-CT either singularly or multiple tibias. The copper tape provides a reference point to identify samples following scanning. (B and C) Growth plates in murine tibias were identified by scrolling through cross sections until solid bone could no longer be seen. (B) Presence growth plate bone can be observed at this cross section. (C) Only trabecular bone can be observed; thus, this section is set as the bottom of the growth plate. (D) Regions of interest were drawn incorporating only trabecular bone and excluding cortical bone. (E and F) Reconstructed 3D imaged of trabecular bone in a sagittal (E) and transverse (F) plane. (G) A region of interest was drawn around the cortical bone, excluding trabecular bone in the center. (H) A region of interest was drawn around the whole bone inclusive of both trabecular and cortical bone present. (I) Reconstructed 3D imaged of cortical bone in a transverse plane. All scale bars 1000 μm.
  b. Place samples into the straws with the growth plate at the top.
  c. Add 70% ethanol into the straw using a pasture pipette, the amount of ethanol needed will depend on the length of the straw used.

***Note:*** When using the three-straw method (Figure 1A**)** you must be able to identify your sample both after the scan and in the scanned image, this can be done using copper tape on one straw that will be detected on the scan and can be used to identify which sample is which in the final image. Ensure that the copper tape is not overlapping with the tibia sample as this will obstruct the tibia scan, due to the density of the copper.

1. Turn on and warm up the SkyScanner1172 micro-CT machine.
2. Log onto a connected PC and load the Skyscanner1172 software (version 1.5).
3. Run flat field correction.
  a. Open the stage and ensure no samples are present.
  b. Set the camera to far position.
      Set the filter to Aluminum 0.5 mm and image resolution to Medium: 2000 x 1200 (Pixel size of 9.00 μm).^5^^,^^6^
  c. Alter acquisition modes:
    i. Press CTRL + ALT + SHIFT + S to unlock special acquisition modes.
    ii. Go to options and select “Acquisition modes”.
    iii. Set X-ray voltage to 60 kV.
      ***Note:*** The voltage is a measure of X-ray energy. A higher voltage allows for the X-ray to penetrate through thicker samples, such as rat bone, without altering the image quality.
    iv. Set X-ray current to 199 μA.
      ***Note:*** Changing the current alters signal to noise ratio. A higher current allows for a better signal, whilst reducing the noise.
    v. Set exposure to 580 ms.
      ***Note:*** Exposure is the amount of time given for the photons to be detected at each scan point. A higher exposure time reduces the noise and increases the signal intensity. However, it will increase the scan time. Exposure should be adjusted to ensure that transmission without a sample is >65%, but the pixels are not maxed out at 100%. Aim for 80–85% transmission.
    vi. Set rotation step to 0.45°, Frame average to 4 and random movement to 0.
  d. Go to Option, Preferences and untick the boxes for flat field correction.
  e. Select acquire Bright + Dark Flat Field Data and click OK.
    ***Note:*** This process should take around 17 min to complete.
4. When finished open options and preferences and re-select flat field correction, that were unselected in 3e.
5. Turn on the X-Ray by pressing the camera button:
  a. Right click the image to bring up contrast levels. If levels are around 80–85% continue, otherwise adjust the exposure and redo step 3.
6. Prepare tibias for scanning:
  a. For scanning samples must be kept upright and still to avoid movement during the scan, to do this a standard straw (diameter ∼6 mm) can be used (Figure 1A).
    i. To allow for multiple samples to be imaged at once, three straws can be placed together (Figure 1A).
    ii. Glue straws into the center of a bijou lid, ensuring they are as central as possible to ensure they are centered in the scanning chamber (Figure 1A).

7. Open the scanner door and place the sample in the middle of the stage as vertical as possible.

***Note:*** If the sample is not vertical it may cause the sample to move out of range when rotating.

8. Select real time (Picture of the TV) to show real time image and right click to check transmission is between 65–75%, this number is lower due to the added density of the sample in the chamber.
9. Run the sample:
  a. Open the options tab and select “data acquisition”:
    i. A dialog box will open.
    ii. Select medium pixel size.
    iii. Rotation step 0.45.
    iv. Frame average 4.
    v. Selecting partial scan will allow you to reduce the size of scan area.
      ***Note:*** if reducing scan area ensure sample remains in scan area, this can be done by rotating the sample stage.
  b. Select a folder for the scans to be saved.
  c. Go to file and select “start acquisition”.
  d. Begin scan.
    ***Note:*** This process should take around 35–40 min per run to complete, but will depend on settings altered during the protocol.

### Reconstruction of micro-CT image

**Timing: 10 min per sample**

Here we describe how to reconstruct micro-CT images, allowing subsequent analysis of trabecular and cortical bone and the generation of 3D constructs.

10. Open GPU-NRecon Server to allow for faster processing of samples.

***Note:*** The GPU-NRecon Server requires a graphics card with the NVidia “CUDA” language enabled. NRecon reconstruction can be run with without the use of the GPU-NRecon server if a graphics card cannot be obtained, however reconstruction will be significantly slower.

11. Open NRecon software.
12. Open a recently scanned TIF image. The whole dataset from that run should open in NRecon.
13. Drag the red lines on the open dataset to choose a range to reconstruct. For analysis of tibias, this will be the whole bone.

***Note:*** If the machine used is different to that specified in this protocol, camera and specimen placement may result in the whole tibia not being visible. This can be overcome by adjusting camera location to a far position if possible or altering scan settings for an “Oversized scan”.

14. Select Settings within the reconstruction menu and ensure the following:
  a. Misalignment compensation is turned on.^7^
  b. Misalignment compensation is below 3 or above −3. The sample may require rescanning if misalignment displayed >3 or <−3.
  c. Ring artefacts reduction is set to 4.
  d. Beam hardening is on and set to 20%.

***Note:*** These settings have been developed by the authors to ensure consistency between scans and experiments, however these settings and can be altered at the researcher’s discretion . For example, misalignment settings between −5 and 5 are acceptable and the NRecon “fine-tuning” option can be used to identify the best ring artifact correction setting for a scan. Misalignment can sometimes be improved in post-processing using the NRecon setting “X-Y alignment with reference scan”. This method may be preferential if repeated scanning is not possible.

15. Select Output within the reconstruction menu and ensure that Scales ON is **NOT** selected.
16. Return to the Start tab and select preview.

***Note:*** The preview will reconstruct the location of the green bar on the image. The green bar can be dragged to the observe different areas of the bone.

17. Return to the Output tab and adjust convert the threshold values to “Log” format by double clicking the histogram. Alter the threshold lines by dragging the upper threshold just beyond the last peak.

***Note:*** To ensure the micro-architecture values can be directly compared between samples the upper threshold should remain the same between data sets.

18. Select browse and create a new folder labeled “Rec” and ensure the file format is TIF (16).

***Note:*** If files are too large, files can instead be saved in BMP(8) format.

19. Go to Start and select start – reconstruction should take 4 min per sample.

***Note:*** The file size will vary dependent on the size of the scan area chosen, when using the three-straw technique the file is approximately 650 MB. If multiple scans are to be analyzed, you can “Add to batch” and later select start batch within the Batch settings.

### Analysis of tibia trabecular bone

**Timing: 1 h per sample**

20. Open CT Analyzer (CTAn)^8^ and one image from the reconstructed scan. This will open the dataset within CTAn in 8-bit format.

***Note:*** If tibias were not scanned in an upright position, the orientation of the sample can be adjusted prior to opening files in CTAn by loading the reconstructed files in the Bruker DataViewer software. Reoriented datasets can be saved as BMP(8) files and loaded in CTAn as above.

21. Within the dataset tab, traverse through the images until you reach the bottom of the growth plate. This is noticeable due to the presence of trabecular bone and loss of solid bone (Figures 1B and 1C).
22. Right click on that frame and select ‘set the top of selection’. Right click and select ‘selection’ and input a bottom Z-position 100 sections below the top selection. Because pixel size varies between machines and settings, the number of frames should be altered accordingly, for example the number of frames to equal a length of 1 mm.
23. Click on the ‘regions of interest’ tab.
24. Using your mouse, manually draw around the trabecular bone, excluding the solid bone observed around the outside of the section (Figure 1D).
25. Scroll to the bottom of the selected Z stack and draw a new region of interest. The software will interpolate between the two regions of interest, however new regions of interest can be drawn in between to ensure only trabecular bone is selected.
26. Click ‘binary selection’ tab and from the histogram menu select ‘from dataset’.
27. Move the lower limit threshold to select real bone (∼100 but will depend on image).
  a. To find the correct threshold move between the ‘regions of interest’ and ‘binary selection’ tabs until the trabecular bone is matched between the two.
  b. Keep the threshold the same between samples.
28. Move to the ‘custom processing’ tab within the Plug-ins menu and select: Threshold, despeckle, despeckle, 3D model and 3D analysis.
  a. Within ‘threshold’ select ‘default levels’ to match the threshold previously set.
  b. Within the first ‘despeckle’^9^ select: remove white speckles, in a 3D space, less than 10 voxels. Apply to region of interest.
  c. Within the second ‘despeckle’ select: remove black speckles, in a 3D space, less than 10 voxels. Appy to region of interest.
  d. Within 3D model select .ply file and apply to Image inside ROI with the Marching Cube 33 Algorithm.
  e. Within 3D analysis select Basic Values and Additional values and select a location to save results.

***Note:*** Before continuing, open preferences (file-preferences) and ensure that nomenclature is set to Bone ASBMR and Unit is set to um or mm.

29. Press play.
30. Once the run has finished, open the .Ply file in a 3D model program such as MeshLab to visualize the bone (Figures 1E and 1F).
31. Open the .txt file in Excel using the Delimited function (Data – Text to Columns) splitting by comma.
  a. The file will contain the outputs of Tissue volume (TV), Bone volume (BV), Percent bone volume (BV/TV), Trabecular thickness (Tb.Th), Trabecular separation (Tb.Sp), Trabecular number (Tb.N) and Trabecular pattern factor (Tb.Pf).

***Note:*** If the preferences within the CTAn software are the units in pixels not μm or mm, values must be converted accordingly, pixel size and units are displayed on the Excel sheet. Once the Excel spread sheet is downloaded the values can be scaled using with pixel size and units, this is not required for BV/TV (%) or other values that are percentages. Conversions should take into account the units (μm^3^ compared to μm) to ensure accurate conversion between pixels and actual size.

### Analysis of tibia cortical bone

**Timing: 1 h per sample**

32. Open CT analyzer (CTAn) and one image from the reconstructed scan. This will open the dataset within CTAn in 8-bit format.
33. Within the dataset tab, traverse through the images until you reach the bottom of the growth plate. This is noticeable due to the presence of trabecular bone and loss of solid bone (Figures 1B and 1C).
34. Right click on that frame and select reference (right click, reference) with settings of an offset of −350 and a height of 100 generating a stack 250 – 350 below the growth plate.

***Note:*** This offset allows for the analysis of cortical bone outside of the main trabecular bone region, allowing for analysis of only cortical bone parameters. Care should be taken to ensure that no trabecular bone is located within the ROI. An alternative method is to analyze a 1 mm portion centered around the midpoint of the bone shaft.

35. Click on the Regions of interest tab.
36. Using your mouse, manually draw around the cortical bone, ensuring trabecular bone is excluded as shown in Figure 1G. Save this region of interest as “cortical_bone.roi”.
37. Remove the region of interest and redraw a second ROI encompassing the whole bone in the same region (Figure 1H). Save the second ROI as “whole_bone.roi”.
38. Click ‘binary selection’ tab and from the histogram menu select ‘from dataset’.
39. Move the lower limit threshold to select real bone (∼100 but will depend on image):
  a. To find the correct threshold move between the ‘regions of interest’ and ‘binary selection’ tabs until the trabecular bone is matched between the two.
  b. Keep the threshold the same between samples.
40. Move to the ‘custom processing’ tab within the Plug-ins menu. Select: Threshold, despeckle, 3D model and 2D analysis.
  a. Within ‘threshold’ select ‘default’ levels to match the threshold previously set.
  b. Within ‘despeckle’ select: remove white speckles, in a 3D space, less than 10 voxels, applied to Region of Interest.
  c. Within ‘3D model’ select ‘.ply file’ and ‘apply to image inside ROI’ with the ‘marching cube 33 algorithm’.
  d. Within ‘2D analysis’ select ‘append summary results to file’ and select a location to save results.
41. Select ‘BATch MANager’ (Batman icon) from the plug-ins pane.
42. Within ‘BatMan’ select ‘add’ and choose a reconstructed TIF file from the dataset being analyzed. This will open the whole dataset converted to 8-bit format.
43. Select ROI and select the cortical_bone.roi.
44. Repeat steps 42 and 43 selecting the whole_bone.roi.
45. Click ‘start’ and allow BatMan to run.
46. One run has finished, the .Ply file can be opened in 3D model programs such as MeshLab to visualize the bone (Figure 1I).
47. Output will be a .txt file that will open in excel using the Delimited function (Data – Text to Columns) splitting by comma.
48. Outputs of interest are ‘mean total cross sectional tissue area’ (T.Ar), ‘mean total cross sectional bone area’ (B.Ar) and ‘cross sectional thickness’ (Cs.Th).
49. Percentage cortical area (%) can be calculated using B.Ar from the output generated using cortical_bone.roi and T.Ar from the output generated using whole_bone.roi by calculating B.Ar/T.Ar∗100.

### 3-point-bend testing of femur

**Timing:2h per sample**

50. Frozen femurs should be cleaned of all tissue using forceps and rehydrated in PBS for at least 2 h.

***Note:*** Tissue left on the bone will interfere with the force profile generated, creating less accurate results.

**Figure 2:**
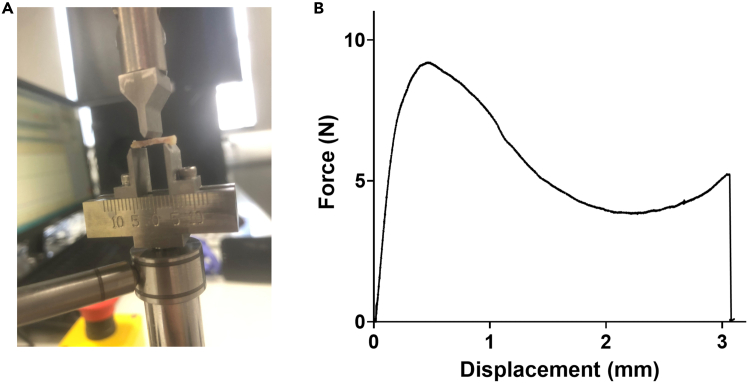
Analysis of bone strength using three-point bend (A) Cleaned femurs were placed on a 22 N load cell with points 8 mm apart on the Distal femur and Trochanter major. (B) Displacement was plotted against Force to enable calculations of stiffness (gradient), load to failure (force needed to form first peak), load at failure (force needed to form second peak), and work required to fracture (area under the curve).
53. Femurs should be placed on the plate ensuring the Distal femur and Trochanter major are balanced.
54. Within the WinTest software package, set the load head to descend at 1 mm/minute, recording force and displacement data.
55. This can be set within the Waveform setup menu and Data Acquisition by selecting Timed Data and Continuous, so 100 data points are recorded every second comprising of time, displacement, and force (N).
56. Pre-load bone (0.2–0.4 N) to prevent rolling during loading.
57. Run the system until the bone fractures, at which point stop the system. This stop can be set automatically using the test limits setting and ensuring the maximal displacement is just above the expected failure value. When the load head moves the maximal distance, it will complete a controlled stop.
58. Within a graphical analysis software (e.g. GraphPad Prism) plot displacement (mm) against force (N) (Figure 2B).
59. This plot can be utilized to calculate stiffness (N/mm; gradient of the rising portion); load to failure (N; maximum load supported by the bone); load to fracture (N; force when the bone no longer bends and snaps) and work required to fracture (N/mm; determined from the area under the curve until fracture).

***Note:*** This methodology can be impacted by bone size. In models where bone size is expected to vary, ensure length is taken before 3-point bending to enable its consideration as a confounding factor.

51. Samples are run on the ElectroForce BioDynamic 5500 (Bose) fitted with a 22 N load cell.
52. The plate should be set 8 mm apart (Figure 2A).

### Calcein analysis of tibiae

**Timing: 3 weeks, analysis 1 h per sample**

60. To embed non-decalcified tibias for sectioning, first dehydrate bone samples at room temperature as follows. Each step should be performed for 48 h:
  a. 70% ethanol.
  b. 80% ethanol x 2.
  c. 90% ethanol x 2.
  d. 100% ethanol x 3.
61. Surround tibias in LR White medium grade resin at 4°C for 6 days, changing to fresh resin every other day.^10^
62. Orientate tibias longitudinally in fresh LR White medium grade resin and polymerize for 16 h at 55°C.
63. Using a microtome, cut 8 μm sections longitudinally at 3 depths 30 μm apart in each tibia.
64. Transfer the sections onto Superfrost Plus slides.
65. Perform imaging analysis using “Osteomeasure” (Osteometrics, Georgia, USA) with an associated fluorescent microscope:
  a. Image the medial and lateral endocortical bone surface, in addition to the trabecular bone in the metaphysis marrow space at 20X magnification.
  b. Measuring 0.3 mm from the growth plate, six fields of view are taken along both the medial and lateral surfaces of the bone for a total distance of 3.6 mm.
  c. Use the average distance between the two calcein labels to calculate mineral apposition rate (MAR) per day calculated by Ir.L.Th/Ir.L.t.
  d. Use the surface area of bone stained with calcein (mineralizing surface; MS) to calculate the percentage of bone mineralizing (MS/BS) by comparison to total bone surface (BS) calculated by 100∗(dL.Pm+ sL.Pm/2)/B.Pm, using endocortical perimeter (Ec.Pm) as B.Pm.
  e. Calculate bone formation rate (BFR) by multiplication of MAR by MS/BS (MAR∗(MS/BS)/100).

### Decalcifying, paraffin embedding, and sectioning bone

**Timing: 21+ days**

66. Make up ethylenediaminetetraacetic acid (EDTA) solution,^11^^,^^12^ protocol for 1 L:
  a. 100 g EDTA added to 700 mL distilled water.
  b. Agitate solution using a magnetic stirrer.
  c. Slowly adjust the pH to 7.4 using 2 M NaOH.
  d. Add supplemental distilled water until the solution volume is 1 L.
  e. Store up to 3 months at 4°C.

***Note:*** Volume required is dependent on the number of samples being processed, one murine sample requires approximately 147 mL. Excess EDTA solution can be stored at 4°C for up to 3 months.

**Figure 3:**
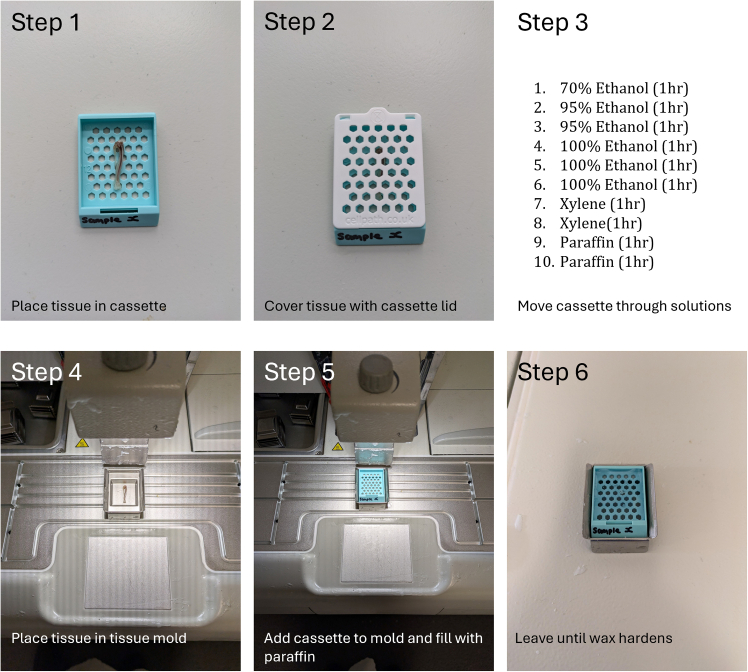
Step by step embedding protocol Murine tibia moved from EDTA solution. washed in PBS and placed in tissue cassette (**step 1**). Lid placed on cassette (**step 2**) and cassette moved through pots containing increasing concentrations of ethanol, xylene or paraffin wax (60°C) (**step 3**). Following dehydration and paraffin infiltration sample moved to a metal tissue mold or the appropriate size (**step 4**) tissue is fixed in place with a small amount of wax. Original tissue cassette from step 1 (blue) is inverted and placed on metal mold as pictured (**step 5**). Mold is filled with paraffin wax and placed on cooling plate until wax hardens (**step 6**).
72. Dehydration of tissue: (Figure 3 – Step 3).
  a. Samples should be moved through at least 10 times volume (i.e. 1 cassette in a minimum of 10 mL) of different containers of increasing concentrations of ethanol:
    i. 70% (1 h);
    ii. 95% (1 h);
    iii. 95% (1 h);
    iv. 100% (1 h);
    v. 100% (1 h);
    vi. 100% (1 h);
  b. Move samples to at least 10 times volume (i.e. 1 cassette in a minimum of 10 mL) of fresh xylene two times:
    i. Xylene (1 h);
    ii. Xylene (1 h);

***Note:*** Caution Xylene is a hazardous substance and must be handled within a chemical fume hood, ensure the user is protected by appropriate PPE (eye goggles, gloves and lab coat).

67. Add 1 fixed and cleaned tibia to 7 mL of EDTA solution in a 15 mL Falcon tube.
68. Place on orbital shaker, ensuring the sample is being agitated constantly at room temperature.
69. Change EDTA solution daily for 3-weeks or until bone mineral within the sample can no longer be detected visually by X-ray (micro-CT). The amount of time required can vary widely between sample types and full decalcification is crucial for successful sections. Always check successful decalcification by X-ray imaging before proceeding.
70. Following decalcification, wash sample once in distilled water at room temperature for 1 min prior to dehydration.
71. If running multiple samples, place each in individual tissue cassettes (Figure 3 – Step 1 & 2).

73. Paraffin infiltration of tissue:
  a. Melt paraffin by heating to 58°C–60°C, the volume of paraffin required is at least 10 times tissue volume, this will alter the melting time required for the wax.
  b. Place samples in at least 10 times volume of melted paraffin wax:
    i. Paraffin (1 h);
    ii. Paraffin (1 h);
74. Embedding of sample:
  a. Add a small amount (approx. 1 mL) of melted paraffin into the bottom of a stainless-steel tissue mold.
  b. Place tissue in the mold and move to desired orientation (Figure 3 – Step 4).
  c. Move mold on to cold plate (−6°C) allowing wax to solidify and fix the tissue in place, this will take approx. 15–30 s.
  d. Place the original cassette inverted on top of the tissue mold (Figure 3 – Step 5).
  e. Fill the tissue mold with wax enough to completely cover the cassette, this will vary dependent on the size of the mold used (Figure 3 – Step 5).
  f. Place on cold plate until wax completely solidifies, time will vary depending on mold size (Figure 3 – Step 6).
  g. Once the wax has hardened remove from mold and store at 4°C for at least 12 h before sectioning.

***Note:*** If wax block is left for too long within metal mold it may be difficult to remove and cause the block to crack.

75. Sectioning:
  a. Prior to sectioning ensure samples are cool and place on a cooling plate or ice.
  b. Using a microtome slice 4 μm sections (Figure 4 – Step 1).

**Figure 4:**
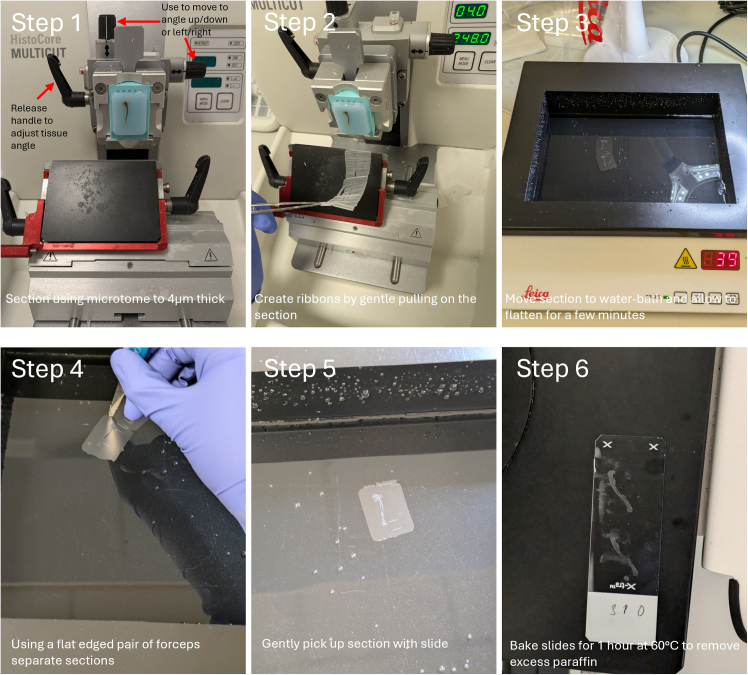
Step by step sectioning guide Block containing tissue is secured into the microtome holder and positioned to allow for sectioning of the whole tissue block by adjusting the microtome head mechanism using the handle indicated in **step 1.** Once positioned begin to section block, by first trimming off the excess wax (10 μm) until the tissue is reached. Once the tissue can be seen move to 4 μm sections. Ribbons can be created by sectioning multiple slices at once, gently pulling on the section (**step 2**). Once a ribbon is created move to 40°C water bath and allow to flatten (**step 3**). Once smooth use blunt headed forceps to separate sections by pinching between two sections (**step 4**). After the section is separated pick up using a slide (**step 5**). Bake section at 60°C for 1 h to remove excess paraffin (**step 6**).
  c. Section multiple slices to create a ribbon (Figure 4 – Step 2).
  d. Place sections in 40°C water bath using tweezers until sections become smooth (Figure 4 – Step 3).
  e. Separate ribboned sections using flat headed forceps (Figure 4 – Step 4).
  f. Use positively charged slides to adhere section (Figure 4 – Step 5) and place in metal slide rack.
  g. Bake slides at 60°C for 1 h to remove wax (Figure 4 – Step 6).
76. Removal of paraffin and rehydration of tissue:
  a. Before staining slides must be deparaffinized using containers of fresh xylene at room temperature in separate pots:
    i. Xylene (5 min);
    ii. Xylene (5 min);
    iii. Xylene (2 min);
  b. Followed by rehydration in decreasing concentrations of ethanol and distilled water in separate pots:
    i. 100% (2 min);
    ii. 90% (2 min);
    iii. 80% (2 min);
    iv. 70% (2 min);
    v. DiH_2_O (2 min);
    vi. DiH_2_O (2 min);
  c. Slides to be kept hydrated in diH_2_O until staining.

### Osteoid staining and analysis

**Timing: 1 h 30 min per sample**

**Figure 5:**
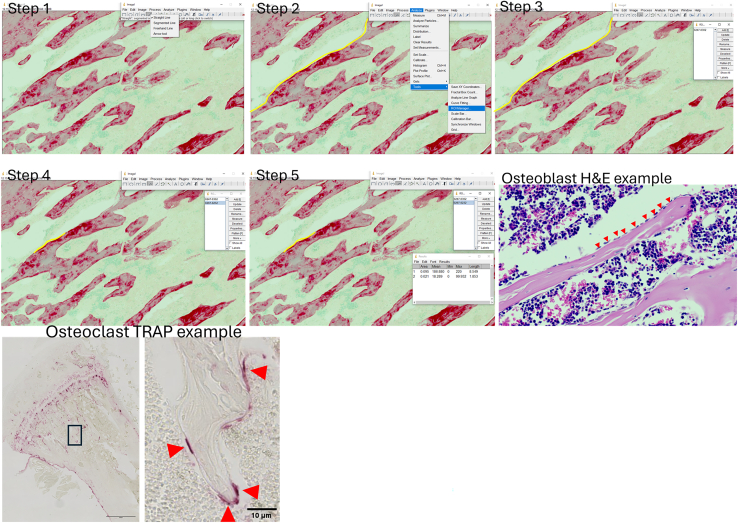
Image analysis tutorial Example of analysis using osteoid stained tibial sections. Freehand line draw tool selected in ImageJ (**step 1**). Line drawn across trabecular bone surface indicated by yellow line in step 2 (line enhanced for visibility). Save ROI to ROI manager located in analysis -> tools -> ROI manager (**step 2/3**). After the trabecular surface ROI is saved draw a new ROI on the osteoid on the same trabecular area as previously measured (**step 4**) and save ROI. Once ROI have been produced, select the ROI for trabecular surface and measure length using ctrl + M, do this for all ROIs. Using the output measurements find the percentage covered trabecular compared to total trabecular surface (**step 5**). Analysis is the same for osteoblast coverage where the trabecular surface covered with osteoblasts, example image in step six, osteoblasts indicated by red arrow. Example of TRAP stained osteoclasts for counting in step 7 with whole stained tibial growth plate (scale bar 100 μm), expanded image (scale bar 10 μm) with osteoclasts indicated by red arrows.
    ii. ROI can be stored using ROI manager (Figure 5 – Step 3).
    iii. Create a new ROI outlining the areas of red staining on the bone surface and save into ROI manager (Figure 5 – Step 4).
    iv. Measure each area using the measure tool by selecting the ROI from the manager and using ctrl + M (Figure 5 – Step 5).
    v. Use measurement to determine percentage of osteoid compared to trabecular bone surface area (OB/OS).^14^^,^^15^

***Note:*** Zen files (.CZI) do not open in all versions of ImageJ. CZI files will open in the Fiji version of ImageJ or if the BioFormat plugin was installed in ImageJ. If required, files must be converted to .tif before ImageJ analysis. When saving images, ensure scale bars are included to allow for length conversion.

77. Staining:
  a. Rehydrated sample slides were stained with 0.04% Fast Green dissolved in distilled water for 30 min.
  b. Wash slides under running water for 1 min.
  c. Counterstain slides using 0.1% Sirius red dissolved in picric acid for 30 min–1 h.^13^
    ***Note:* Caution:** Picric acid is a hazardous substance**: Fire, blast or projection hazard; increased risk of explosion if desensitizing agent is reduced. Harmful if swallowed. Toxic in contact with skin or if inhaled.** Ensure proper PPE is used when handling picric acid, including gloves, safety coat and protective goggles and use in a contained and functioning fume hood.
  d. Wash slides with distilled water until water runs clear.
  e. Dehydrate samples in increasing concentrations of ethanol and xylene in separate pots:
    i. 70% (2 min);
    ii. 95% (2 min);
    iii. 95% (2 min);
    iv. 100% (2 min);
    v. Xylene (5 min);
    vi. Xylene (2 min);
  f. Allow samples to dry for 2 min and mount using DPX mountant and a cover slip.
78. Imaging and analysis:
  a. Image slides using the ZEISS Axioscan Z1 and Zen blue. Images taken using colored bright-field setting using x20 magnification objective (Bright-field imaging, Objective: 20x/0.8 Plan Apochromat, Camera: Hitachi HV-F203 3 CCD, 1600x1200 pixels, 4.4 μm pixel size).
  b. Analyze slides using free ImageJ software:
    i. ROI created of trabecular bone using freehand line drawing tool to measure bone surface area (Figure 5 – Step 1 & 2).

### Osteoblast staining and analysis

**Timing: 30 min per sample**

79. Make before starting staining:
  a. 0.2% ammonia water.
80. Staining:
  a. Rehydrated tissues should be stained with hematoxylin solution for 3 min.
  b. Wash in running tap water for 5 min.
  c. Differentiate in 1% acid alcohol for 30 sec.
  d. Wash in running tap water for 1 minute.
  e. Put in 0.2% ammonia water in a small p10 sized tip box for 1 minute.
  f. Wash in running tap water for 5 min.
  g. Rinse in 95% alcohol, 10 dips.
  h. Counterstain in eosin (aqueous) for 5 min.
  i. Dehydrate samples in increasing concentrations of ethanol and xylene:
    i. 70% (2 min);
    ii. 95% (2 min);
    iii. 95% (2 min);
    iv. 100% (2 min);
    v. Xylene (5 min);
    vi. Xylene (2 min);
  j. Allow samples to dry for 2 min and mount using DPX mountant and a cover slip.
81. Imaging and analysis:
  a. Image slides using the ZEISS Axioscan Z1 and Zen blue. Images taken using colored bright-field setting using x20 magnification objective (Bright-field imaging, Objective: 20x/0.8 Plan Apochromat, Camera: Hitachi HV-F203 3 CCD, 1600x1200 pixels, 4.4 μm pixel size).
  b. Analyze slides using free ImageJ software:
    i. ROI created around trabecular bone using freehand line drawing tool to measure bone surface area (Figure 5 – Step 1 & 2).
    ii. ROI can be stored using ROI manager (Figure 5 – Step 3).
    iii. Create a new ROI outlining the areas of bone lined with multiple cuboidal cells,^16^ example in Figure 5, save to ROI manager (Figure 5 – Step 4).
    iv. Measure each area using the measure tool by selecting the ROI from the manager and using ctrl + M (Figure 5 – Step 5).
    v. Osteoblast surface presented as percentage bone osteoblast coverage (Ob.S/BS).^14^^,^^15^

***Note:*** Zen files (.CZI) do not open in all versions of ImageJ. CZI files will open in the Fiji version of ImageJ or if the BioFormat plugin was installed in ImageJ. If required, files must be converted to .tif before ImageJ analysis. When saving images, ensure scale bars are included to allow for length conversion.

### Osteoclast staining and analysis

**Timing: 1 h per sample**

Osteoclasts number can be analyzed using, tartrate-resistant acidic phosphatase (TRAP) staining.

82. Make TRAP basic incubation medium.

**Table:** TRAP Basic Incubation medium

Reagent	Final concentration	Amount
Sodium acetate anhydrous	112.1 mM	9.2 g
L- (+) Tartaric acid	76 mM	11.4 g
distilled water	N/A	900 mL
Glacial acetic acid	46.65	2.8 mL
5 M Sodium hydroxide	N/A	-
**Total**	**N/A**	**1000 mL**

**Note**: Store at room temperature up to conditions 6 months.

***Note:*** Please consult product safety data sheet prior to making solution.

L- (+) Tartaric acid: **Corrosive** – avoid contact with eyes by wearing tight fitting protective goggles, if substance gets in eyes: Rinse cautiously with water for several minutes. Remove contact lenses, if present and easy to do. Continue rinsing Immediately call a POISON CENTER or doctor/physician.

Acetic acid (Glacial): **Flammable liquid and vapor. Causes severe skin burns and eye damage**. Ensure correct PPE is used, gloves, safety coat and protective goggles.

5 M Sodium hydroxide: **May be corrosive to metals ad can cause sever skin burns and eye damage**. Ensure correct PPE is used, gloves, safety coat and protective goggles and use in a well-ventilated area.

83. Dissolve and adjust pH to 4.7–5.0 with 5 M Sodium hydroxide to increase or more Glacial acetic acid to decrease pH.
84. Bring total volume to 1 L.
85. Make up TRAP Staining Solution.

**Table:** 

Reagent	Final concentration	Amount
TRAP Basic Incubation medium	N/A	50 mL
Napthol AS-MX	1.4 mM	20 mg
2-Ethoxyethanol	221.92 mM	1 mL
Fast Red Violet LB salt	1.59 mM	30 mg
**Total**	**N/A**	**1000 mL**

**Note**: Make up fresh before each stain.

***Note:*** Please consult product safety data sheet prior to making solution.

2-Ethoxyethanol: **Flammable liquid and vapor. Harmful if swallowed. Toxic if inhaled. May damage fertility. May damage the unborn child.** Ensure correct PPE is used, gloves, safety coat and protective goggles and use in a well-ventilated area. Contact a doctor/physician if ingested or inhaled.

Fast Red Violet LB salt: **Suspected of causing cancer.** Ensure correct PPE is used, gloves, safety coat and protective goggles and use in a well-ventilated area.

86. Dissolve 20 mg Napthol AS-MX in 1 mL 2-Ethoxyethanol in a 1.5 mL Eppendorf and vortex until powder completely dissolved.
87. Add 50 mL TRAP basic incubation medium to a 50 mL falcon tube and add 1 mL of Napthol AS-MX mixture.
88. Add 30 mg fast Red Violet LB salt to TRAP solution and vortex until completely dissolved.
89. Warm solution for 10 min at 37°C before use.
90. Staining:
  a. Place deparaffinized/rehydrated slides in pre-warmed TRAP staining solution mix and incubate at 37 ͦC for 30 min – 2 h until the staining has developed.
    ***Note:*** The time taken for staining to develop can vary. Observe slides every 10 min under a light microscope. This staining could cause cells to come up as a red/violet color, however if left too long the entire tissue will stain pink. Once stained cells are visible, remove from the staining solution.
  b. Once stain has developed rinse in distilled water.
  c. Remove excess water using a paper towel.
  d. Mount slides in aqueous mounting media and a cover slip.
    ***Note:*** This staining procedure can also be used for cultured osteoclasts in vitro. For staining, cells should be fixed with 10% PFA for 10 min and washed with three times PBS. After washing trap solution can be added to cover cells and incubate at 37 ͦC for 30 min, checking periodically to prevent over staining.
91. Imaging and Analysis:
  a. Image slides using the ZEISS Axioscan Z1 and Zen blue. Images taken using colored bright-field setting using x20 magnification objective (Bright-field imaging, Objective: 20x/0.8 Plan Apochromat, Camera: Hitachi HV-F203 3 CCD, 1600x1200 pixels, 4.4 μm pixel size).
  b. Analyze slides using free ImageJ software:
    i. ROI created around trabecular bone using freehand line drawing tool to measure bone surface area.
    ii. Measure each area using the measure tool by selecting the ROI from the manager and using ctrl + M.
    iii. ROI can be stored using ROI manager.
    iv. Using the counting tool count the number of TRAP positive osteoclasts present on the bone surface.
    v. Osteoclast number presented as number of osteoclasts per mm^2^ (OC.N/BS).^14^

***Note:*** Zen files (.CZI) do not open in all versions of ImageJ. CZI files will open in the Fiji version of ImageJ or if the BioFormat plugin was installed in ImageJ. If required files must be converted to .tif before ImageJ analysis. When saving images, ensure scale bars are included to allow for length conversion.

***Note:*** Due to the faint staining of TRAP, automated microscopes such as the slide scanner can struggle to detect samples. To combat this, draw an ROI in black pen around the sample.

## Expected outcomes

This protocol allows for analysis of murine long bones to understand the structure of trabecular and cortical bone (micro-CT analysis), femur strength (3-point-bend), bone growth (sectioning with osteoid and calcein analysis) and cellular composition (sectioning with osteoblast H&E staining). Overall, these outcomes can be combined to generate an in-depth overview of bone, which can be analyzed following changes in disease states or in response to novel therapeutics.

## Limitations

The thresholding of bone requires manual adjustment. If scanning settings are maintained between samples, the threshold should be maintained between samples. However, if scanning settings differ (e.g. due to machine and x-ray source ageing), care should be taken when adjusting the threshold to ensure only real bone is selected. Additionally, all settings included here are specific to male C57BL/6J mice between 8–12 weeks of age. Depending on mouse strain, sex, age and disease state, these microCT settings must be adjusted accordingly. However, settings should not be changed within an experiment, as this could alter the levels of bone detected and bias results.

Three-point bend testing is also limited in understanding the mechanics of bone, due to the load being placed on a specific section of the bone, causing the fracture at one point. This can be adjusted by using 4-point bending, where the weakest point of the bone should fracture.

Manual error can also be made when identifying the growth plate. If regions containing the growth plate are erroneously included within the trabecular bone region of interest, it will result in increased trabecular bone measurements due to incorporation of the growth plate in calculation. If the ROI is set too far below the growth plate, trabecular bone will be missed, resulting in lower measurements. To avoid bias, it is recommended that experiments are blinded throughout the analysis stage and that two independent researchers carry out the micro-CT analysis for more consistent results.

## Troubleshooting

### Problem 1

The bone can spin within the straw as the μCT base plate rotates, resulting in blurring and artifacts in the image.

### Potential solution

Place piece of polystyrene on top of sample to prevent bone movement, the polystyrene will not be detected by the μCT and will not interfere with imaging.

### Problem 2

Difficulty sectioning paraffin blocks.

### Potential solution

There are multiple reasons for sectioning to be compromised, therefore it is important to check different aspects of the processing method:

• Decalcification, if tissue is not properly decalcified samples will be difficult to section to ensure decalcification has been successful check with μCT. Reasons for improper decalcification include:
  ○ Volume of EDTA not enough for size of tissue – ensure there is at least 10 times the volume of EDTA solution as there is tissue, we find 7 mL per tibia is suitable but if issues occur increasing volumes will improve this.
  ○ Length of time is not enough for complete decalcification – EDTA is a chelating agent and removes calcium from the bone, this takes longer than other decalcification methods but maintains better tissue integrity compared to other decalcifying agents such as formic acid. To speed up this process changing the EDTA solution daily prevents saturation of the EDTA solution with calcium increasing calcium leaching.
• Processing, if the tissue is processed incorrectly prior to embedding it can lead to difficulties when sectioning:
  ○ Heat damage to sample – avoid leaving tissue in paraffin wax for more time than required.
  ○ Not enough volume of liquid – increase volumes of ethanol, xylene and paraffin, especially when processing multiple samples.
• Embedding, if cracks or air bubbles form within wax blocks sections may not cut evenly and result sections tearing. If this occurs the block must be melted down and re-embedded. Reasons which may cause wax cracking:
  ○ Initial wax over hardened before filling section – only allow to set for 5 sec.
  ○ Block cooled too quickly – increase temperature of cold plate or allow to harden at room temperature.

Sectioning paraffin-embedded bone tissue can be tricky despite proper tissue processing, therefore we have outlined a few tips which may improve sectioning.

• Ensure block is cooled prior to sectioning –
  ○ As with other tissues it is important that the wax is cooled before processing, we have found the best way to do this is keep tissue blocks in the fridge overnight.
  ○ One hour before cutting, remove an ice block from the freezer and leave at room temperature. This will slightly thaw the ice preventing cracking of the blocks when they are placed on ice.
  ○ Once the ice is slightly thawed, place the blocks face down on the ice. If there are more than 5 blocks, we recommend removing them from the fridge in groups as opposed to all blocks sitting on ice.
  ○ After sitting on ice for about 15 min the blocks are ready to be sectioned.
  ○ Between sections always place the block back on ice – a sign that this is required is that sections will begin to wrinkle, and the block will become more difficult to cut.
• Ensure blade is sharp –
  ○ When sectioning bone the blade becomes dulled more quickly than with other soft tissues. Move along the blade regularly during sectioning and replace blade when there is no fresh blade remaining.

• Alter orientation and size of bone tissue –
  ○ The region of long bone which is most likely to break when sectioning is the diaphysis, which can result in tissue fragmentation or complete section loss .
  ○ Bone breakage can be caused by processing (see above for tips) or by the angle in which the bone is embedded and sectioned.
  ○ In this protocol we orientate the long bone so it is in a vertical position. When sectioning, the blade passes though the lower metaphysis and epiphysis into and through the diaphysis prior to the upper metaphysis and epiphysis. This allows the whole bone to be sectioned, however as the blade is passing though regions of different densities it is not uncommon for the tissue to break.
  ○ There are two ways that this can be combatted depending on the experimental question:
    - If the experimental question requires only the growth plate, reducing the length of the bone sample will reduce the chance of tissue breakage. This also allows for use of a smaller wax mold.
       Artifacts introduced during the tissue cutting process must be taken into consideration during analysis.
    - If the experimental question requires the whole bone, orientation of the tissue horizontally may help.

### Problem 3

Over staining using Sirius red/H&E.

### Potential solution

There are multiple reasons why the staining might be over exposed:

• Paraffin not completely removed causing the stain to cling to paraffin – ensure paraffin is completely removed by increasing baking length (avoiding over baking the tissue) or increasing the length of time in xylene.
• Sections under decalcified – for Sirius red staining differences in levels of decalcification can affect staining, therefore ensure decalcification protocol is kept constant. If overstaining persists decrease staining length.

## Resource availability

### Lead contact

Further information and requests for resources and reagents should be directed to and will be fulfilled by the lead contact, Helen McGettrick (h.m.mcgettrick@bham.ac.uk).

### Technical contact

Further technical information on the experiments and protocols should be directed to and will be fulfilled by the technical contacts, Amy Naylor (a.naylor@bham.ac.uk), Jonathan Lewis (j.lewis.2@bham.ac.uk), or Kathryn Frost (kathryn.e.frost00@gmail.com).

### Materials availability

This study did not generate new unique reagents.

### Data and code availability

This study did not generate any unique datasets or codes.

## Acknowledgments

J.W.L., K.F., and G.N. were supported by PhD studentships funded by Medical Research Council – Versus Arthritis Centre for Musculoskeletal Ageing Research PhD studentship (MR/R502364/1) and British Society for Research on Ageing – Chernajovsky Foundation PhD scholarship. A.J.N. was supported by a Versus Arthritis Career Development Fellowship (21743). This work was also supported by a Medical Research Council project grant MR/T028025/1 and the UK SPINE Knowledge Exchange Network. This paper represents independent research funded in part by the MRC – Versus Arthritis Centre for Musculoskeletal Ageing Research (MR/P021220/1) and the Research into Inflammatory Arthritis Centre Versus Arthritis (RACE) (grant number 22072). The views expressed are those of the author(s) and not necessarily those of the MRC or Versus Arthritis.

Analysis was conducted using the Imaging Suite funded by the University of Birmingham, and the micro-CT facility within the “Science City Research Alliance” was funded by Advantage West Midlands and the European Regional Development Fund. Dynamic histomorphometry analysis was performed by members of the Skeletal Analysis Laboratories (The University of Sheffield).

## Author contributions

Conceptualization and design, A.J.N. and H.M.M.; acquisition of data, J.W.L., K.F., G.N., H.A., and E.P.; analysis and interpretation of data, J.W.L., K.F., and G.N.; resources, supervision, and project administration, A.J.N. and H.M.M.; writing – original draft, J.W.L., K.F., and H.M.M.; writing – reviewing and editing and approving the final version of the manuscript, all authors.

## Declaration of interests

The authors declare no competing interests.
